# *CHD2* haploinsufficiency is associated with developmental delay, intellectual disability, epilepsy and neurobehavioural problems

**DOI:** 10.1186/1866-1955-6-9

**Published:** 2014-04-22

**Authors:** Sébastien Chénier, Grace Yoon, Bob Argiropoulos, Julie Lauzon, Rachel Laframboise, Joo Wook Ahn, Caroline Mackie Ogilvie, Anath C Lionel, Christian R Marshall, Andrea K Vaags, Bita Hashemi, Karine Boisvert, Géraldine Mathonnet, Frédérique Tihy, Joyce So, Stephen W Scherer, Emmanuelle Lemyre, Dimitri J Stavropoulos

**Affiliations:** 1 Division of Medical Genetics, Department of Pediatrics, Centre Hospitalier Universitaire de Sherbrooke, 3001, 12E Avenue Nord, Sherbrooke, QC J1H 5N4, Canada; 2 Division of Clinical and Metabolic Genetics, Department of Pediatrics, The Hospital for Sick Children and University of Toronto, 555 University Ave, Toronto, ON M5G 1X8, Canada; 3 Department of Medical Genetics, Alberta Children’s Hospital Research Institute, University of Calgary, 2888 Shaganappi Trail NW, Calgary, AB T3B 6A8, Canada; 4 Division of Medical Genetics, Department of Pediatrics, Centre Hospitalier Universitaire de Québec, 2705 Boulevard Laurier, Québec, QC G1V 4G2, Canada; 5 Cytogenetics Department, Guy’s and St Thomas’ NHS Foundation Trust, Great Maze Pond, London SE1 9RT, UK; 6 Department of Molecular Genetics and McLaughlin Centre, The Centre for Applied Genomics and Program in Genetics and Genome Biology, The Hospital for Sick Children and University of Toronto, 686 Bay Street, Toronto, ON M5G 0A4, Canada; 7 Division of Anatomic Pathology and Cytopathology, Cytogenetics Laboratory, Calgary Laboratory Service and Alberta Children’s Hospital, 2888 Shaganappi Trail NW, Calgary, AB T3B 6A8, Canada; 8 Division of Medical Genetics, Department of Pediatrics, Centre Hospitalier Universitaire de Sainte-Justine, Université de Montréal, 3175, Chemin de la Côte-Sainte-Catherine, Montréal, QC H3T 1C5, Canada; 9 Department of Clinical Genetics, Lakeridge Health Oshawa, 1 Hospital Court, Oshawa, ON L1G 2B9, Canada; 10 Department of Paediatric Laboratory Medicine, The Hospital for Sick Children and University of Toronto, 555 University Avenue, Toronto, ON M5G 1X8, Canada

**Keywords:** Autism spectrum disorder, *CHD2*, Developmental delay, Epilepsy, Learning disability

## Abstract

**Background:**

The chromodomain helicase DNA binding domain (CHD) proteins modulate gene expression via their ability to remodel chromatin structure and influence histone acetylation. Recent studies have shown that CHD2 protein plays a critical role in embryonic development, tumor suppression and survival. Like other genes encoding members of the CHD family, pathogenic mutations in the *CHD2* gene are expected to be implicated in human disease. In fact, there is emerging evidence suggesting that *CHD2* might contribute to a broad spectrum of neurodevelopmental disorders. Despite growing evidence, a description of the full phenotypic spectrum of this condition is lacking.

**Methods:**

We conducted a multicentre study to identify and characterise the clinical features associated with haploinsufficiency of *CHD2*. Patients with deletions of this gene were identified from among broadly ascertained clinical cohorts undergoing genomic microarray analysis for developmental delay, congenital anomalies and/or autism spectrum disorder.

**Results:**

Detailed clinical assessments by clinical geneticists showed recurrent clinical symptoms, including developmental delay, intellectual disability, epilepsy, behavioural problems and autism-like features without characteristic facial gestalt or brain malformations observed on magnetic resonance imaging scans. Parental analysis showed that the deletions affecting *CHD2* were *de novo* in all four patients, and analysis of high-resolution microarray data derived from 26,826 unaffected controls showed no deletions of this gene.

**Conclusions:**

The results of this study, in addition to our review of the literature, support a causative role of *CHD2* haploinsufficiency in developmental delay, intellectual disability, epilepsy and behavioural problems, with phenotypic variability between individuals.

## Background

Chromatin remodeling is the dynamic modification of chromatin architecture essential to many biological processes, including cell division, gene expression, and DNA replication, packaging and repair [1-3]. The chromodomain helicase DNA binding (CHD) proteins belong to the SNF2-related superfamily of ATPases, which use the energy from ATP hydrolysis to change nucleosome positioning, composition and chromatin structure. The CHD family is defined by the presence of tandem chromo (chromatin organisation modifier) domains in the N-terminal region and a central SNF2-related helicase/ATPase domain [4]. The latter is the catalytic core mediating chromatin alteration. Members of the CHD family are divided into three subfamilies according to their additional structural motifs. (1) CHD1 and CHD2 possess a C-terminal DNA binding domain recognizing AT-rich DNA motifs. (2) CHD3 and CHD4 contain a pair of plant homeodomain zinc finger domains in their N-terminal regions and lack a DNA binding domain. (3) CHD5 to CHD9, which contain diverse additional functional domains such as the BRK (Brahma and Kismet) domain, SANT-like (switching-defective protein 3, adaptor 2, nuclear receptor corepressor, transcription factor IIIB) domain, CR domain and DNA binding domain [1,5].

As CHD proteins play pivotal roles in modulating chromatin structure and are involved in processes such as gene activation and repression, DNA recombination and repair, cell-cycle regulation, development and cell differentiation, dysregulation of these proteins may have adverse effects on human development. Heterozygous mutations of the *CHD7* gene are known to cause the multisystem abnormalities associated with autosomal dominant CHARGE syndrome (coloboma, heart anomaly, choanal atresia, retardation, genital and ear anomalies) [OMIM:214800]. Characteristic anomalies include ocular coloboma, choanal atresia, cranial nerve defects, distinctive external and inner ear abnormalities, hearing loss, cardiovascular malformations, intellectual disability (ID), urogenital anomalies and growth retardation. More recently, exome sequencing studies have shown loss of function mutations affecting one allele of *CHD8* to be associated with autism spectrum disorder (ASD) [6-8].

There is emerging evidence showing *CHD2* haploinsufficiency is associated with neurodevelopmental abnormalities. Deletions affecting this gene are very rare, and, so far, there is only one case report of a child with global developmental delay, epilepsy and ASD who was found to have a *de novo* deletion encompassing the *RGMA* and *CHD2* genes [9]. However, because of the importance of *RGMA* in central nervous system axonal growth, haploinsufficiency of this gene was thought to be a good candidate to explain the patient’s developmental delay and seizures. A deletion affecting *CHD2* was also reported in the supplemental data derived from genomic microarray analysis of 996 patients ascertained to have ASD [10]. This deletion involves *CHD2*, but not *RGMA*, and the patient was reported to have ASD and mild ID with no history of seizures or language delay. Recent studies have implicated *de novo* intragenic sequence mutations in *CHD2* in individuals with ID and a range of epileptic encephalopathies [11-13]. Although there is increasing evidence suggesting that mutations in *CHD2* contribute to a broad spectrum of neurodevelopmental disorders, a description of the full phenotypic spectrum is lacking.

In order to investigate the role of *CHD2* haploinsufficiency in neurodevelopmental disorders, we describe the first series of patients with deletions affecting *CHD2* from among a cohort of 42,313 patients broadly ascertained by clinical genetics laboratories to have developmental delay, intellectual disability, multiple congenital anomalies and/or ASD. We also present a review of the literature to correlate our patients’ phenotypes with those previously reported with deletions and loss-of-function sequence mutations affecting this gene.

## Methods

We conducted a multicentre study of retrospective genomic copy number variation (CNV) data from six genetic diagnostics laboratories (see Additional file 1: Table S1), to collect phenotypic information from patients with deletions affecting *CHD2*. For all patients, phenotypic information was collected from genetics clinic assessments and medical chart reviews. This study is compliant with the research ethics boards of each participating institution. Signed informed consent was obtained from all study participants or their legal representatives. DNA extracted from uncultured cells, typically from peripheral blood lymphocytes, was used to perform genomic CNV analysis in all participating laboratories. Microarray experiments were performed according to the manufacturer’s instructions. As described in Table S1, the microarray platforms used at each of the six diagnostic centres were oligonucleotide array–based. Deletions were confirmed, and parental follow-up studies were performed with multiplex ligation-dependent probe amplification (MLPA), array comparative genomics hybridization (aCGH) or fluorescence *in situ* hybridization (FISH) analysis. FISH analysis was performed using fosmid G248P83477D10 or CTD-2314 N10 bacterial artificial chromosome. We investigated the frequency of deletions affecting *CHD2* in high-resolution CNV data from among 13 control cohorts comprising a total of 26,826 individuals [10,14-26].

## Results

**Figure 1:**
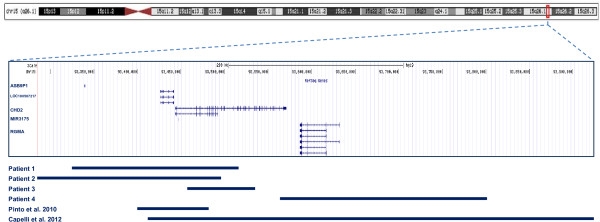
Summary of deletions observed in our patients and reported in the literature.

We reviewed genomic CNV data from six genetic diagnostics laboratories that perform microarray analysis for patients broadly ascertained to have delayed developmental milestones in motor, speech and/or cognition skills (developmental delay); diagnosis of global developmental delay or ID, multiple congenital anomalies; and/or ASD. We identified four deletions affecting *CHD2* exonic sequences from a total of 42,313 patients analyzed. The deletions ranged in size from approximately 78 kb to 237 kb, with only one case also involving the *RGMA* gene, and all were found to be *de novo* (Figure 1). No other clinically significant CNVs were found in these patients. The clinical findings in these patients included developmental delay, learning difficulties or ID in all four patients, as well as seizures in three patients (Table 1). The dysmorphic features observed in each of these patients are described in turn in the subsections below. However, no characteristic facial gestalt was found. Brain magnetic resonance imaging (MRI) did not reveal any malformations in our patients. Analysis of data derived from 26,826 individuals from population-based control cohorts evaluated by high-resolution CNV analysis did not show deletions affecting exonic sequences of *CHD2*, confirming the extreme rarity of this deletion in the general population (see Additional file 1: Table S2).

**Table 1:** **Clinical characteristics of patients with *CHD2 *** **deletions**^**a**^

**Characteristics**	**Patient 1**	**Patient 2**	**Patient 3**	**Patient 4**	**Pinto *et al*.**[10]	**Capelli *et al*.**[9]
Gender	F	F	F	F	M	F
Age (yr)	11	9	6	16	Not specified	6
Chr15 deletion [hg19] start–end (bp)	93,324,047 to 93,515,100	93,286,333 to 93,496,391	93,456,168 to 93,534,338	93,563,564 to 93,800,894	93,399,003 to 93,482,000	93,412,860 to 93,923,856
Size	191 kb	210 kb	78 kb	237 kb	83 kb	511 kb
RefSeq genes	*CHD2*, *ASB9P1*, *LOC100507217*, *MIR3175*	*CHD2*, *ASB9P1*, *LOC100507217*, *MIR3175*	*CHD2*	*CHD2*, *RGMA*	*CHD2*, *LOC100507217*, *MIR3175*	*CHD2*, *RGMA*, *LOC100507217*, *MIR3175*
Inheritance	*De novo*	*De novo*	*De novo*	*De novo*	*De novo*	*De novo*
Development	Motor delay		Communication disorder (receptive and expressive language difficulties)	Globally delayed	Globally delayed with more significant speech delay	Unknown	Globally delayed
	Speech delay						Speech impairment
	Cognition	Learning disability	Learning disability	ID	ID	ID	Unknown
		Short-term memory problems	Short-term memory problems				
			Visual perceptual disability				
	Behaviour	Short attention span	ADHD	Aggressive, impulsive, repetitive behaviours	ASD	ASD	Autistic behaviour
			Limited social skills		Aggressive behaviour		
		Aggressive behavior					Short attention span
		Limited social skills					
Seizure type (age of onset)	Jeavons syndrome	Absence seizures (3 yr)	No epilepsy	Complex partial and generalised seizures	No epilepsy	Unspecified seizures (2 yr)	
	Absence seizures						
	Eyelid myoclonia (6 yr)						
Brain MRI	Normal	Not done	Normal	Normal	Altered angular gyrus	No severe abnormalities	
Other	Mild hypotonia	Mild hypotonia	Mild hypotonia	Tourette’s syndrome			Gait ataxia
		Feeding difficulties	Feeding difficulties				Slight hypotonia
Dysmorphic features	Square-shaped face	Triangular face	Brachycephaly			Protruding ears	Facial gestalt suggestive of Angelman syndrome
			Prominent forehead	Broad forehead		Micrognathia	
		High forehead		Short nose, upturned tip			
			Full lips				
		Prominent columella	Widely spaced central maxillary incisors				Wide mouth
							Widely spaced teeth
	Short philtrum						
	Fifth-finger brachydactyly	Micrognathia					Prognathia
	Syndactyly of toes 2 and 3						
Other features	Mild thoracic scoliosis	Reduced body fat mass	Strabismus	Mild thoracic scoliosis		Strabismus	
	PIP joint fusion of thumbs						
	Mild peripheral hearing loss (higher frequencies)						
	Duplex kidney						

^a^ADHD*,* Attention-deficit hyperactivity disorder; ASD, Autism spectrum disorder; Chr, Chromosome; ID, Intellectual disability; MRI, Magnetic resonance imaging; PIP, Proximal interphalangeal; RefSeq, National Center for Biotechnology Information (NCBI) Reference Sequence Database (http://www.ncbi.nlm.nih.gov/refseq/).

### Patient 1

Patient 1 has a 191-kb *de novo* deletion involving *CHD2*, the *ASB9P1* noncoding pseudogene, the uncharacterised *LOC100507217* noncoding gene and microRNA *MIR3175* (Figure 1). This 11-year-old girl was referred for medical genetics evaluation because she had fine and gross motor delays as well as seizures. She was born by Caesarean section at 38 weeks gestation with a birth weight of 2,977 g (25th percentile) to healthy nonconsanguineous Caucasian parents. She sat upright at age 1 year, walked at 22 months and spoke her first words at 3 years. She had learning disabilities with specific deficits in language and mathematics, and she repeated grade 3. She also had short-term memory problems, a short attention span, poor social skills and aggressive behaviour. She developed seizures at 6 years of age, which were characterised by absence seizures with eyelid myoclonia (Jeavons syndrome) and confirmed by video electroencephalogram (EEG). Her medications included lamotrigine and ethosuximide. She also had auditory neuropathy consistent with bilateral mild loss of peripheral auditory function at higher frequencies. Her physical examination revealed that she had subtle dysmorphic features, with a square-shaped face, high forehead, short philtrum, thin upper vermillion border of the lip and prominent columella. She had mild thoracic scoliosis, fusion of the proximal interphalangeal joint in both thumbs, bilateral fifth-finger brachydactyly, tapered fingers and cutaneous 2-3 syndactyly of the toes. Her neurological examination was normal, with the exception of frequent episodes of eyelid fluttering bilaterally. An MRI scan taken when she was 9 years of age was normal, as was a skeletal survey; however, an abdominal ultrasound showed a duplex right kidney. She had a normal acylcarnitine profile and normal plasma amino acids, electrolytes, lactate, urinary organic acids and FRAXA and FRAXE fragile X syndrome tests.

### Patient 2

Patient 2 is a 9-year-old girl who has a 210-kb deletion affecting *CHD2*, the *ASB9P1* noncoding pseudogene, the uncharacterised *LOC100507217* noncoding gene and microRNA *MIR3175* (Figure 1), similar to patient 1. She was referred to medical genetics for mild delays in motor and language development as well as seizures. She was born at term (41 weeks gestation) after an uneventful pregnancy to nonconsanguineous parents. Her birth weight was 3,610 g (50th percentile). She was found to have mild axial hypotonia as an infant. She first walked at age 15 months. She had feeding problems in infancy. She was diagnosed with attention-deficit/hyperactivity disorder and was being treated with dextroamphetamine and amphetamine. She showed limited social skills without any other features associated with ASD. She had visual perceptual disabilities, a communication disorder characterised by mixed receptive and expressive language difficulties and short-term memory problems. She repeated grade 1, and her performance on the Wechsler Intelligence Scale for Children–Fourth Edition was classified in the Low Average range for her age group. Absence seizures that began at 3 years of age were being treated with levetiracetam and valproic acid. Her physical examination showed that she had reduced body fat mass, a prominent forehead, a triangular face, full lips, widely spaced maxillary central incisors and micrognathia. Her neurological examination was normal. Investigations performed for the assessment of her seizures and developmental delays included G-banding karyotype, a metabolic screen including carnitine level and acylcarnitine profile, plasma amino acids, and urinary organic acids, all of which were normal. An EEG showed generalised epileptogenic dysfunction and photosensitivity. No brain imaging was performed.

### Patient 3

Patient 3 has a *de novo* 78-kb intragenic deletion affecting several 5′ exons of *CHD2*. No other gene was deleted in this patient. This 6-year-old girl was referred to the clinical genetics clinic for assessment of her profound delay in motor development and hypotonia. Her mother’s pregnancy was unremarkable, and she was born by Caesarean section at term with a birth weight of 2,920 g (25th percentile) to healthy nonconsanguineous parents. She had feeding difficulties as an infant and had ongoing issues with chewing and swallowing food properly in early childhood. She was found to have mild hypotonia in infancy, but she had normal tone at age 6 years. She sat upright at 9 months of age and walked at 26 months. At 6 years of age, she was not able to climb stairs with alternating feet and had difficulty pedaling a tricycle. She had mild delays in her fine motor skills, and, although she had normal speech, her language comprehension skills were delayed. She scored below the first percentile on the Wechsler Preschool and Primary Scale of Intelligence–Third Edition full-scale IQ test at 4 years of age. She displayed repetitive behaviours, such as walking in circles, mouthing objects and head-banging, but she did not meet the criteria for ASD. She had a history of aggressive behaviour towards others, which has mostly resolved. She was described as having lack of insight and judgment, as well as impulsive behaviour. She had never had seizures, and her EEG and brain MRI were normal. Her medical history is significant for strabismus. Her physical examination revealed that she had normal growth parameters, including a normal head circumference. Her facial features included brachycephaly, a broad forehead with a short nose, and an upturned nasal tip. Her physical examination was otherwise unremarkable. Molecular genetic testing showed normal results for methylation-specific MLPA analysis for Prader-Willi and Angelman syndromes, *MECP2* sequencing and FRAXA fragile X syndrome testing.

### Patient 4

Patient 4 was found to have a 237-kb *de novo* deletion involving *CHD2* and *RGMA*. Analysis by aCGH in this 16-year-old girl was performed because of poorly controlled epilepsy as well as ASD. She is known to have very challenging behavioural issues and Tourette’s syndrome. She presented in childhood with delays in motor, speech and cognition, with a more significant delay in language. At school, she exhibited difficulties in spelling and reading skills and had difficulty with mathematics. Her IQ ranged between 35 and 49. Complex partial and generalised seizures were noted between 3 and 24 months of age. Her physical examination showed that she had normal growth parameters with mild thoracic scoliosis. No striking dysmorphic features were reported. Her brain MRI was normal.

## Discussion

We present the clinical features of four individuals with heterozygous *de novo* deletions affecting *CHD2* who were identified from among a phenotypically heterogeneous cohort of 42,313 patients with developmental delays or ID, multiple congenital anomalies and/or ASD. The clinical symptoms of our patients are consistent with and include delays in speech and/or motor development, seizures, ID and/or learning difficulties, and neurobehavioural abnormalities, which may include autistic features, ADHD and/or aggressive behaviour. Our examination of 26,826 individuals from 13 control cohorts did not show any deletion affecting exonic sequences of this gene, indicating that deletions affecting *CHD2* are extremely rare.

Our review of the literature on *CHD2* deletions turned up one case report describing a *de novo* deletion affecting *CHD2* and *RGMA* in a patient with speech and motor delays, including ID, gait ataxia, dysmorphic features, autistic features with attention deficit, and seizures beginning at 24 months of age [9]. These features were attributed to haploinsufficiency of *RGMA* and/or *CHD2*. In our cohort, only one patient had a heterozygous deletion affecting *CHD2* and *RGMA*. Nevertheless, all of our patients had similar clinical findings, suggesting that *CHD2* contributes significantly to the broad spectrum of neurodevelopmental disorders and mild dysmorphic features seen in patients with *CHD2* deletions (Table 1). In addition, in an examination of the supplemental data from genomic CNV analysis of an ASD cohort, Pinto *et al*. [10] reported a *de novo* deletion of *CHD2* in one patient with mild ID and ASD but no seizures [10]. This suggests that epilepsy may not always be present in patients with *CDH2* haploinsufficiency, as we observed in one of our four patients. In another report in the literature, Kulkarni *et al*. [27] described a *de novo* translocation t(X;15)(p22.2;q26.1)dn disrupting *CHD2* in a child with developmental delay, scoliosis and hirsutism. It is possible, however, that the clinical presentation of their patient may have been affected by disrupted expression of other genes near the translocation breakpoints. Interestingly, two of our patients with *CHD2* deletions had mild thoracic scoliosis, suggesting that *CHD2* disruption may predispose individuals to vertebral anomaly, as reported by Kulkarni *et al*. [27] and as described in the *Chd2*-mutant mouse model [5,27]. Together, the consistent clinical features among patients diagnosed by routine clinical microarray and the *de novo* occurrence of all deletions affecting *CHD2* reported thus far support a causative role of *CHD2* haploinsufficiency for developmental delay, intellectual disability, epilepsy and behavioural problems, with phenotypic variability among individuals (Table 1).

The results of recent studies in which researchers used massively parallel sequencing in patient cohorts investigated for epilepsy, ASD or ID provide supporting evidence for a role of *CHD2* haploinsufficiency in manifestation of the characteristics observed in our patients (Table 2). Carvill *et al*. [11] performed sequence analysis of 66 candidate genes in 500 patients clinically diagnosed with epileptic encephalopathy. They found mutations predicted to be pathogenic in four patients with heterozygous *de novo* nonsense mutations and in two patients with *de novo* missense mutations disrupting the highly conserved residues in the SNF2-related helicase/ATPase domain. They described all six of their patients as having moderate to severe ID in addition to the onset of myoclonic seizures by 3 years of age. A role for *CHD2* in ID is further substantiated by a report of a *de novo* frameshift mutation identified by exome sequencing in one patient [12]. In another report, an exome sequencing screen carried out for *de novo* mutations in patients with infantile spasms and Lennox-Gastaut syndrome revealed one patient with a *CHD2* missense mutation [13]. More recently, Suls *et al*. [28] found three patients carrying *de novo CHD2* sequence mutations who had febrile seizures followed by therapy-resistant generalised seizures (Table 2). In addition, they showed that a knockdown of *chd2* in zebrafish resulted in clinical and electrographic seizures [28]. Interestingly, none of our four patients with a deletion of *CHD2* had febrile seizures. These previous studies, together with our present case series, provide strong evidence that haploinsufficiency of *CHD2* is associated with neurodevelopmental disabilities. Despite the presence of mild dysmorphic features in most patients described thus far, no specific facial gestalt has yet been reported. In addition, no brain malformation has been visualised by MRI. Although massively parallel sequencing studies suggest a strong association between *CHD2* haploinsufficiency and seizures or ID, our study, which is based on broadly ascertained clinical cohorts, shows that such associations are not always observed. Some phenotypic variability between individuals seems to be present. Future studies will reveal whether other genetic factors influence the phenotype of this disorder or if *CHD2* haploinsufficiency is associated with other phenotypes.

**Table 2:** **Clinical characteristics of patients with *CHD2 *** **single-point DNA mutation**^**a**^

**Characteristics**	**Rauch *et al*.**[12]	**Carvill *et al*.**[11]						**Allen *et al*.**[13]	**Suls *et al*.**[28]		
Gender	F		M	F	F	M	M	M	M	M	F	M
Age (yr)	5.75	17	12	29	12	15	2.5	Unknown	6	24	6	
Protein change (type)	p.Thr604 Leufs*19 (frame shift)	p.Glu1412 Glyfs*64 (frame shift)	p.Arg121* (frame shift)	p.Gly491 Valfs*13 (frame shift)	p.Arg1644 Lysfs*22 (frame shift)	p.Trp548Arg (missense)	p.Leu823Pro (missense)	c.1502+1 G>A (splice donor)	c.1810-2A>C (p.?)	c.4971G>A (p.Trp1657*)	c.1396C>T (p.Arg466*)	
Inheritance	*De novo*	*De novo*	*De novo*	*De novo*	*De novo*	*De novo*	*De novo*	*De novo*	*De novo*	*De novo*	*De novo*	
Development	Globally delayed	Mild delay	Normal prior to epilepsy	Unspecified delay	Normal before seizure onset	Unspecified delay	Unspecified delay	Unspecified delay	Normal prior to epilepsy	Normal prior to epilepsy	Subtle motor and speech delay	
Cognition	Mild ID	Moderate ID	Severe ID	Severe ID	Severe ID	Moderate ID	Severe ID	Unknown	Moderate ID	Moderate ID	Mild ID	
	Behaviour	Uncontrolled behavioural anomalies	ASD Behavioural problems	Unknown	Unknown	Unknown	Unknown	ASD	Unknown	Unknown	Unknown	ASD
												ADHD
Seizure type (age of onset)	AS (5 yr)	AtS (1 yr), AS, FS, MJ, MJ-AS, TC	MJ (1 yr), MA, NCS, TC, TS	Atypical AS (1 yr), AtS, LGS, MJ, NCS, SE, TC, TS	AtS (2 yr), MJ, SE, TC	TC (3 yr), FDS, H, MJ	MJ (2.5 yr), FDS, MJ, MA, TS	TC (6 mo), Atypical AS, AtS, FDS, LGS, MS	FS (14 mo), Atypical AS, MS, SE, TC	FS (2 yr), MA, MS, TC	TC (3.5 yr), Atypical AS, AtS, FS, H, MS	
Brain MRI	Unknown	Unknown	Unknown	Unknown	Unknown	Unknown	Unknown	Normal	Normal	Normal	Nonspecific atrophy	
Other									Right hemibody weakness	Dysarthria		Mild ataxia
										Ataxia		
Other features	Duane anomaly								Language regression after corpus callosotomy			

^a^AS, Absence seizure; ASD, Autism spectrum disorder; AtS, Atonic seizure; FDS, Focal dyscognitive seizure; FS, Febrile seizure; H, Hemiclonic; ID, Intellectual disability; LGS, Lennox-Gastaut syndrome; MA, Myoclonic absence; MJ, Myoclonic jerk; MRI, Magnetic resonance imaging; MS, Myoclonic seizure; NCS, Nonconvulsive status; SE, Status epilepticus; TC, Tonic-clonic; TS, Tonic seizure.

## Conclusions

Herein we describe the first series of patients with deletions affecting *CHD2*. We provide additional evidence that deletions and pathogenic point mutations affecting *CHD2* are associated with neurodevelopmental problems, which include delays in speech and/or motor development, seizures, ID or learning difficulties, minor dysmorphic features and behaviour problems involving social difficulties and maladaptive behaviours. Although haploinsufficiency of *CHD2* is associated with a broad spectrum of neurodevelopmental disorders, we show that variability in the clinical expression of the phenotype can be observed. The overview of all currently reported mutations and their associated phenotypic features provided in this study provides a valuable resource for health-care providers caring for individuals with *CHD2* mutations.

## Abbreviations

aCGH: Array comparative genomics hybridization; ADHD: Attention-deficit/hyperactivity disorder; ASD: Autism spectrum disorder; CHD: Chromodomain helicase DNA binding domain; CNV: Copy number variation; EEG: Electroencephalogram; FISH: Fluorescence *in situ* hybridization; ID: Intellectual disability; MLPA: Multiplex ligation-dependent probe amplification.

## Competing interests

The authors declare that they have no competing interests.

## Authors’ contributions

SC, EL and DJS conceived of this study and participated in its design. SC, GY, BA, JL, ACL, CRM, JWA, AKV, SWS, CMO and DJS performed data accumulation and interpretation and participated in manuscript preparation. RL, AKV, KB, GM, FT and JS participated in data accumulation. SC, DJS, GY, ACL, CRM, AKV, BH, JS and SWS participated in critically revising the manuscript. All authors read and approved the final manuscript.

## Supplementary Material

Additional file 1: Table S1 Number of patients tested and microarray platform used by genetic diagnostics laboratories. **Table S2.** Control cohorts examined for exonic deletions at *CHD2*. Click here for file
