# Supplementary material for: CHD2 haploinsufficiency is associated with developmental delay, intellectual disability, epilepsy and neurobehavioural problems
Source: J Neurodev Disord. 2014 Apr 22;6(1):9. doi: 10.1186/1866-1955-6-9 (PMC4022362; doi:10.1186/1866-1955-6-9)
Supplement: Additional file 1: Table S1 — Number of patients tested and microarray platform used by genetic diagnostics laboratories. Table S2. Control cohorts examined for exonic deletions at CHD2. [file 1866-1955-6-9-S1.doc]

**Supplimental Tables**

Table S1. Number of patients tested and microarray platform used by genetic diagnostic laboratories

| **Genetic diagnostic laboratory** | **# Patients Tested** | **Microarray platform** |
| --- | --- | --- |
| The Hospital for Sick Children, Toronto | 7970 | ISCA 180K array, Oxford Gene Technology |
| Alberta Children’s Hospital, Calgary | 2063 | CytoChip ISCA 60K array, BlueGnome. v2.0 |
| King’s College London, London | 17848 | Agilent 60K array |
| Centre Hospitalier Universitaire Sainte-Justine, Montréal | 9411 | NimbleGen CGX-12 135K array, Signature Genomics. v1.0 |
| Credit Valley Hospital, Mississauga | 4144 | CytoChip ISCA 60K arry, BlueGnome. v2.0 |
| Centre Hospitalier Universitaire de Sherbrooke, Sherbrooke | 877 | CytoSure ISCA 180K array, Oxford Gene Technology. V2.0 |
| **Total** | **42313** |  |

Table S2. Control cohorts examined for exonic deletions at *CHD2*

| **Control dataset** | **# Samples** | **Microarray platform** | **# CHD2 deletions** | **Description of control cohort** |
| --- | --- | --- | --- | --- |
| Ontario ARCTIC | 1,120 | Affymetrix 500k | 0 | Zogopoulos (2007) |
| POPGEN | 1,123 | Affymetrix 6.0 | 0 | Krawczak (2006) |
| Ottawa Heart Institute controls | 1,234 | Affymetrix 6.0 | 0 | Stewart (2009) |
| HapMap (Phase 3) | 1,056 | Affymetrix 6.0 | 0 | Altshuler (2010) |
| Starr County Diabetes study | 1,794 | Affymetrix 6.0 | 0 | Below (2011) |
| Geneva NHS/HPFS Diabetes study | 5,966 | Affymetrix 6.0 | 0 | Qi (2010) |
| Ontario Population Genomics Platform (OPGP) | 416 | Affymetrix 6.0 | 0 | Costain (2013) |
| Wellcome Trust (WTCCC) controls | 4,783 | Affymetrix 6.0 | 0 | Craddock (2010) |
| SAGE consortium controls | 1,287 | Illumina 1M | 0 | Bierut (2010) |
| Health, Aging, and Body Composition (Health ABC) Study | 2,566 | Illumina 1M-Duo | 0 | Coviello (2012) |
| KORA | 1,775 | Illumina Omni2.5M quad | 0 | Verhoeven (2013) |
| COGEND | 1,213 | Illumina Omni2.5M quad | 0 | Bierut (2010) |
| Itsara et al. controls | 2,493 | Multiple | 0 | Itsara (2009) |
| **Total** | **26,826** |  | **0** |  |
